# Label-free blood serum detection by using surface-enhanced Raman spectroscopy and support vector machine for the preoperative diagnosis of parotid gland tumors

**DOI:** 10.1186/s12885-015-1653-7

**Published:** 2015-10-05

**Authors:** Bing Yan, Bo Li, Zhining Wen, Xianyang Luo, Lili Xue, Longjiang Li

**Affiliations:** 1 Department of Otolaryngology Head and Neck Surgery, the First Affiliated Hosipital of Xiamen University, Xiamen, China; 2 State Key Laboratory of Oral disease, Sichuan University, Chengdu, Sichuan China; 3 College of Chemistry, Sichuan University, Chengdu, Sichuan China; 4 Department of Stomatology, the First Affiliated Hosipital of Xiamen University, Xiamen, China

**Keywords:** Parotid gland tumor, SERS, SVM, Preoperative diagnosis, Nanoparticle

## Abstract

**Background:**

It is difficult for the parotid gland neoplasms to make an accurate preoperative diagnosis due to the restriction of biopsy in the parotid gland neoplasms. The aim of this study is to apply the surface-enhanced Raman spectroscopy (SERS) method for the blood serum biochemical detection and use the support vector machine for the analysis in order to develop a simple but accurate blood serum detection for preoperative diagnosis of the parotid gland neoplasms.

**Methods:**

The blood serums were collected from four groups: the patients with pleomorphic adenoma, the patients with Warthin’s tumor, the patients with mucoepidermoid carcinoma and the volunteers without parotid gland neoplasms. Au nanoparticles (Au NPs) were mixed with the blood serum as the SERS active nanosensor to enhance the Raman scattering signals produced by the various biochemical materials and high quality SERS spectrum were obtained by using the Raman microscope system. Then the support vector machine was utilized to analyze the differences of the SERS spectrum from the blood serum of different groups and established a diagnostic model to discriminate the different groups.

**Results:**

It was demonstrated that there were different intensities of SERS peaks assigned to various biochemical changes in the blood serum between the parotid gland tumor groups and normal control group. Compared with the SERS spectra of the normal serums, the intensities of peaks assigned to nucleic acids and proteins increased in the SERS spectra of the parotid gland tumor serums, which manifested the differences of the biochemical metabolites in the serum from the patients with parotid gland tumors. When the leave-one-sample-out method was used, support vector machine (SVM) played an outstanding performance in the classification of the SERS spectra with the high accuracy (84.1 % ~ 88.3 %), sensitivity (82.2 % ~ 97.4 %) and specificity (73.7 % ~ 86.7 %). Though the accuracy, sensitivity and specificity decreased in the leave-one-patient-out cross validation, the mucoepidermoid carcinoma was still easier to diagnose than other tumors.

**Discussion:**

The specific molecular differences of parotid gland tumors and normal serums were significantly demonstrated through the comparison between the various SERS spectra.But compared with the serum SERS spectra reported in the other studies, some differences exist between the spectra in this study and the ones reported in the lietratures. These differences may result from the various nano-particles, the different preparation of serum and equipment parameters, and we could need a further research to find an exact explanation.Based on the SERS spectra of the serum samples, SVM have shown a giant potential to diagnose the parotid gland tumors in our preliminary study. However, different cross validaiton methods could effect the accuracy and a further study involing a great number of samples should be needed.

**Conclusions:**

This exploratory research demonstrated the great potential of SERS combined with SVM into a non-invasive clinical diagnostic method for preoperative diagnosis of parotid gland tumors. And the internal relation between the spectra and patients should be established in the further study.

## Background

Among the neoplasms arising in the salivary glands, the parotid gland tumors are the most common with a frequency about 80 % [1, 2]. The accurate preoperative diagnosis of the parotid gland tumors is very essential and important clinically, because the discrimination between the benign and malignant tumors influences the following management of the optimal surgical procedures [2, 3]. Unfortunately, the routine biopsy is not recommended in the parotid gland tumors due to the possibility of the implantation metastasis and facial nerve injury [2]. Although the fine needle aspiration biopsy is considered as a well-established diagnostic technique in the preoperative diagnosis of parotid gland tumors, it can still lead to some defects and limits such as hematoma and bacterial infection. Meanwhile, the result of fine needle aspiration biopsy could be influenced by the experiences of the operator and pathologist [2, 4–6]. So it is important and essential to develop a new technique to make an accurate and noninvasive preoperative diagnosis of parotid gland tumors.

Raman spectroscopy is a vibrational spectroscopic technique based on the inelastic scattering, which can reflect the molecular structures and changes of samples and considered as the molecular ‘fingerprint’ [7–9]. Due to the technical advances in the Raman spectroscopy system instruments and application of multivariate analytic algorithm, the potential use of Raman spectroscopy has been widely developed in tumor diagnostics [2, 10, 11]. Compared with other optical diagnostic technique, Raman spectroscopy owns the advantages such as non-invasive, high spatial resolution, weak water scattering and no sample preparation [10, 12, 13]. However, the signal of Raman spectrum produced by a smaller number of photons (approximately 1 in 10^6^ to 1 in 10^8^) is very weak and covered by a strong autofluorescence background in some cases [14]. These drawbacks hindered the further clinical application in tumor diagnosis. In order to overcome these drawbacks, the increased excitation laser power and collection time were used, but these resulted in the changes and damages of biochemical samples. So the surface-enhanced Raman spectroscopy (SERS) is developed and becomes a promising solution. The SERS is based on the effect of surface-enhanced Raman scattering that the signal of Raman scattering can be greatly enhanced by 10^5^–10^14^ when the molecules are absorbed onto nanostructured metal or colloid surfaces [16–18]. Because of the increased Raman scattering cross-section and high sensitivity, SERS can overcome the drawback of regular Raman spectroscopy and is applied successfully in the diagnosis and discrimination of various tumors [16, 18, 19].

The successful use of Raman spectroscopy in the *ex vivo* diagnosis of the parotid gland tumors has been reported in our previous studies [2, 20]. Due to the interference of the superficial skin and subcutaneous tissues, the Raman spectroscopy of parotid gland tumors cannot be obtained successfully in our *in vivo* study. Previous studies have shown that the plasma or serum levels of RNA, DNA and other biochemical materials changed in patients with cancer and the SERS of peripheral blood, plasma or serum could be used to detect the presence of cancer with a high sensitivity and specificity [16, 18, 21, 22]. So the SERS of peripheral blood could provide an opportunity for non-invasive preoperative diagnosis of parotid gland tumors. In this study, we firstly developed the method of blood serum detection by using SERS based on label-free Au nanoparticles to diagnose the parotid gland tumors preoperatively. The support machine vector is applied to analyze the differences between the SERS data and discriminate the patients from healthy subjects.

## Methods

### Subjects and protocol

In this study, all the patients with the parotid gland tumors were divided into the pleomorphic adenoma (PA) group, the Wartin’s tumor (WT) group and mucoepidermoid carcinoma (MEC) group. Then the patients with old maxillofacial fracture and healthy volunteers were selected as the normal control group. The more detailed information on the subjects was shown in Table 1. All the subjects in this study were not treated prior to this study, didn’t have any other systemic diseases or drug abuse, and their blood routine and biochemistry examinations were all in the normal range. The final diagnoses of patients with the parotid gland tumors were carried out by two experienced pathologists after the surgical operation according to the World Health Organization histological criteria [23]. All the subjects participated in this study were informed detailedly and gave their written informed consent at the beginning of the study. This study was approved by the Institution Review Board of West China Hospital of Stomatology and followed the guidelines of the Helsinki Declaration.

**Table 1:** Information on these subjects in this study

Information	Group			
	PA	WT	MEC	Normal
Age (year)				
Age range	28–68	35–74	29–79	18–65
Median age	41	59	43	35
Gender				
Male	8	17	10	20
Female	12	4	9	11
Total	20	21	19	31

### Blood serum samples

After 10 h of overnight fasting, a single 5 ml peripheral blood sample was obtained from the subjects at 8:00 A.M. without any anticoagulant. Then the blood sample was deposited at 4 °C for 4 h and centrifuged at 3400 rpm (2722 g) for 10mins in order to remove the blood cells, fibrinogen and platelet. After the centrifugation, the blood serum was obtained and 1 ml supernatant was collected as the serum sample and stored at −20 °C for Raman measurement.

### Preparation of Au NPs

**Fig. 1:**
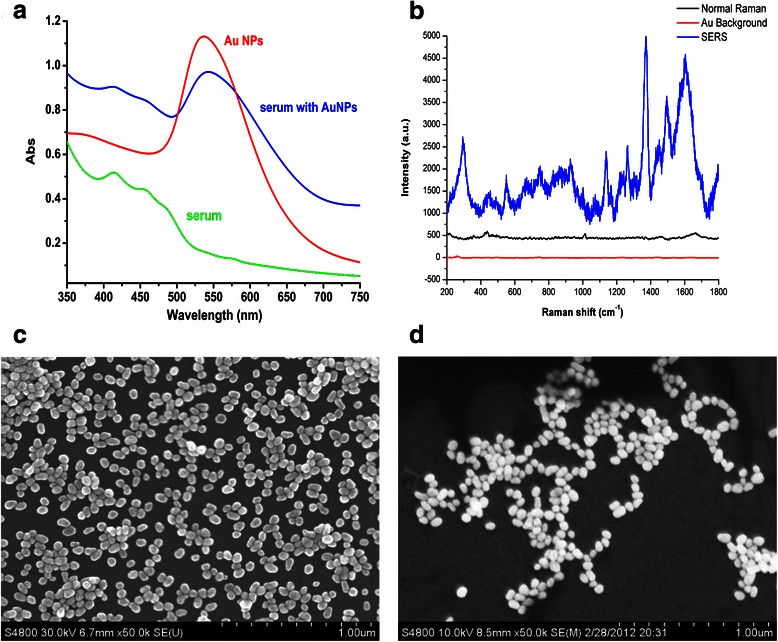
**a** The UV/visible absorption spectra of the Au NPs, serum and the mixture of Au NPs and serum. **b** SERS spectrum of serum, normal Raman spectrum of serum and background Raman spectrum of the Au NPs colloid. **c** The SEM images of Au NPs. **d** The SEM images of the mixture of Au NPs and serum

Au NPs were prepared through the deoxidizing process according to the method reported by Frens [24]. A beaker of 100 ml of 0.1 g/L HAuCl_4_ was heated to the rolling boil and then added the 0.7 ml of 10 g/L sodium citrate rapidly. The mixed solution was heated to keep boiling and stirred continuously for 30mins. During this process, the color of the solution changed from pale yellow to burgundy due to the production of Au NPs. The nanoparticle form of the Au NPs prepared in the above method was spherical with a mean diameter of 55 nm and the UV-visible absorption spectrum shown a significant absorption at 550 nm (Fig. 1).

### SERS measurement

A 4 ml Au NPs solution was centrifuged at 6000 rpm (3219 g) for 10mins,discarded the supernatant and added a 0.4 ml serum sample for the SERS measurement. Before the SERS measurement, the mixed solution was incubated at 4 °C for 2 h. Then a drop of the mixed solution was transferred onto the coverslip for the SERS detection. The confocal Raman micro-spectrometer system (Renishaw, Great Britain) was employed for the SERS measurement of the serum sample using a 633 nm excitation laser. The excitation laser with a power of approximately 0.4 W was focused on 4–6 regions of each drop for the SERS spectral record through a × 50 objective lens (NA = 0.75). The spectra were recorded in the 200–1800 cm^–1^ Raman shift range with a 2 cm^−1^ spectral resolution and a 10s integration time.

### Data analysis

The raw spectral data were preprocessed by WiRE 2.0 software (Renishaw, Great Britain) before the Data statistical analysis in order to remove the interference noises and oversaturated spectra. The LABSPEC 2.0 software (HORIBA Scientific, France) was utilized to remove the autofluorescence background by the 4th polynomial function and smooth the SERS spectra by the Savitzky-Golay smoothing. And all the smoothed SERS spectra were normalized in the region of 200–1800 cm^−1^.

Then the preprocessed data were put into the OriginPro8.0 software(OringinLab, USA) to calculate and produce the mean spectrum of each group. The comparison between the spectra of parotid gland tumors group and normal group were made through the subtraction of different mean spectra and the shifts of the different peaks in the subtracted spectral were assigned to the molecular structures and biochemical component based on the previous studies and literatures [15–18, 25–31] (Table 2).

**Table 2:** Raman shifts of peaks and the characteristic assignments [15–18, 25–31]

Raman shift (cm^−1^)	Peak assignment
292–296	Au-S band
543–548	S-S disulfide stretching in Proteins
723–727	Hypoxanthine
744–747	Thymine in DNA
933	C-C stretching mode, C-C αhelix in proteins
1084	C-C stretching mode in phospholipids
1094	C-N stretching mode in D-Mannos
1127	C-C stretching in lipids, C-N stretching in D-Mannos
1140	Carotenoids
1261–1264	CH bending in lipids
1326–1329	CH vibration in DNA/RNA, CH_2_ twisting in lipids
1368–1373	Guanine in DNA, Tryptophan in proteins
1441–1445	CH_2_, CH_3_ bending in proteins and lipids
1541–1551	C-N stretching, Amide II
1607	C = C band in Phenylalanine or Tyrosine
1698–1699	Amide I

In the process of training SVM, the Gaussian radial basis function was selected and the Jackknife method was employed to optimize the penalty parameter C and kernel-related parameter gamma [20]. Then in the process of testing SVM, the leave-one-sample-out and leave-one-patient- out methods were utilized to test the prediction performance of the diagnostic model established in the process of training SVM. And when the leave-one-patient-out method was applied, all spectra from one patient were left out to test the prediction. Due to the classification of tumor and normal samples, the process of discrimination and diagnosis was divided into two steps as reported in the previous literature [20]. First, the SVM was employed to discriminate the tumor groups from the normal group. Then, the tumor group were classified and diagnosed by SVM respectively. In order to value the efficiency of the model developed by SVM, the employed parameters were as following: the specificity (SP), sensitivity (SE), accuracy (ACC), Matthew correlation coefficient (MCC) and rigidity (R).

## Result

### SERS spectra

In the UV-visible absorption spectra shown as Fig. 1a, the pure serum samples absorption band appeared in the around 420 nm wavelength region and the band of Au NPs appeared in the around 550 nm wavelength region. When the Au NPs were mixed with the serum, the intense of their assigned absorption bands reduced and the shapes of peaks changed. This result was believed to originate from the localized surface plasmon resonance of Au NPs deposited on the biochemical substances in serum [15, 28]. Compared with the normal Raman spectrum, there was a dramatic increase in the intensity of serum SERS, which was resulted from the surface enhance effect produced by Au NPs [Fig. 1b]. The SEM images of the pure Au NPs and the mixture of Au NPs and serum were shown in the Fig. 1c and d respectively. The SEM image showed the conjunction of Au NPs and biochemical substances in serum.

**Fig. 2:**
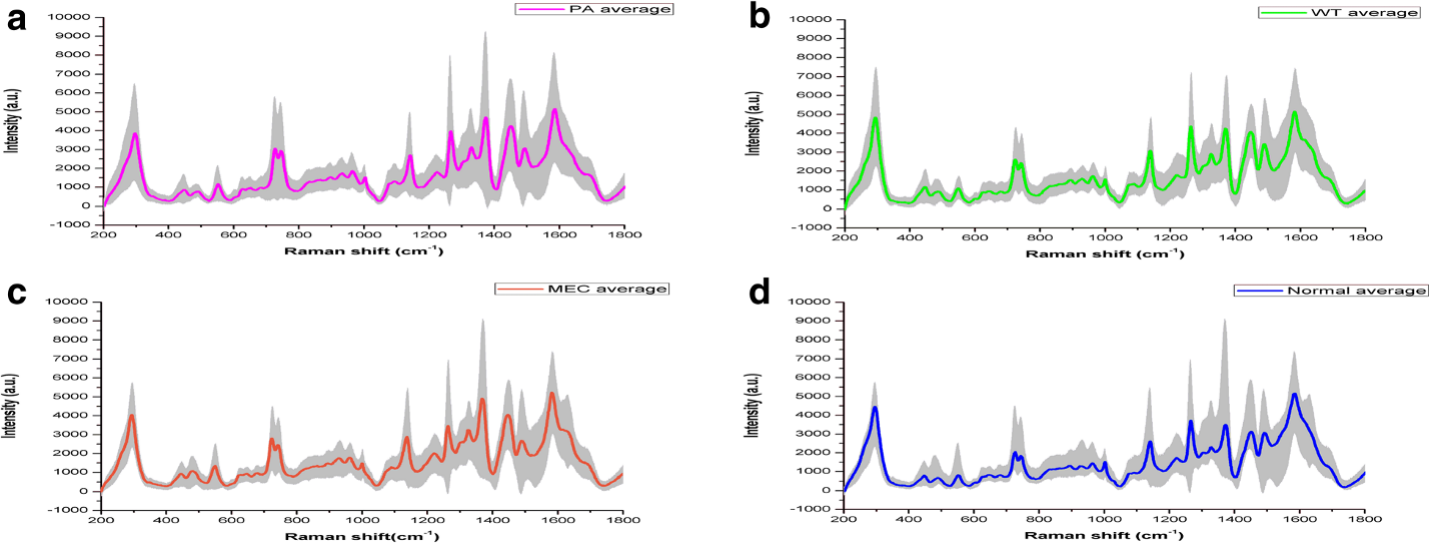
The average Raman spectra of PA, WT, MEC and normal control samples. (**a**: The average spectrum of PA. **b**: The average spectrum of WT. **c**: The average spectrum of MEC. **d**: The average spectrum of normal samples). The gray areas manifest the standard deviations

**Fig. 3:**
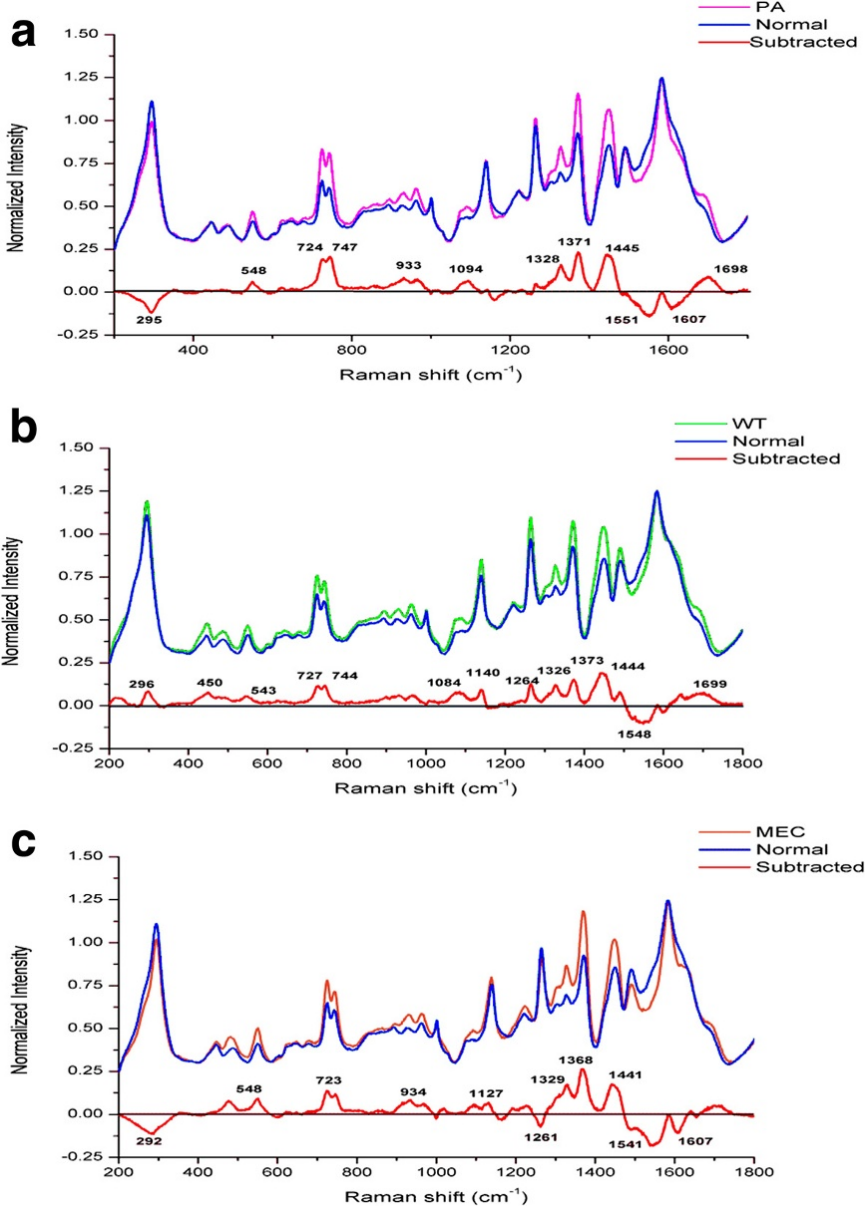
**a** Comparison of the PA and normal group. **b** Comparison of the WT and normal group. **c** Comparison of the MEC and normal group

After the SERS measurement, a total of 454 SERS spectra were obtained successfully from the 91 serum samples, including 101 spectra of PA samples, 105 spectra of WT samples, 95 spectra of MEC samples and 153 spectra of normal samples. The mean SERS spectra of different samples before the spectral normalization were shown in the Fig. 2. Then compared with the normalized mean SERS spectra of the normal groups, the PA groups showed the increase in the peaks at 548, 724, 747, 933, 1094, 1328, 1371, 1445, 1698 cm^−1^ but the decrease in the peaks at 295, 1551, 1607 cm^−1^, which was shown in the subtracted spectrum in Fig. 3a. There were also differences in the mean SERS spectra between the normal groups and the WT groups, the subtracted spectra in Fig. 3b showed the increase in the peaks at 296, 450, 543, 727, 744, 1084, 1140, 1264, 1326, 1373, 1444 and 1699 cm^−1^ but the only decrease in the peak at 1548 cm^−1^. The subtracted spectrum from the MEC and normal groups showed the increase in the peaks at 548, 723, 934, 1127, 1329, 1368 and 1441 cm^−1^ but the decrease in the peaks at 292, 1261, 1541 and 1607 cm^−1^, which was shown in the Fig. 3c. All these peaks can be assigned to different biochemical components and molecular structures such as nucleic acids and proteins, and in order to better understand the different peaks in the subtracted spectra, Table. 2 lists the characteristic assignments of peaks at different Raman shifts based on the previous literatures and studies. In the parotid tumors groups, compared with the normal groups, the intensities of peaks at the 720–750 cm^−1^ and 900–1450 cm^−1^ regions increased, which were assigned to the molecular structures in the nucleic acids, proteins and lipids, but the intensities of peaks at the around 1500–1600 cm^−1^ region decreased, which were assigned to some special molecular bond or vibration. The assignments of major peaks can be shown in Table 2. And based on these differences of peaks, the spectra of different tumors and normal groups can be classified and diagnosed.

### SVM diagnosis

In order to develop effective diagnostic algorithms for differentiation between the parotid tumor groups and normal group, the process of SVM diagnosis contains two steps. The first step was to discriminate the normal samples from the parotid tumor samples respectively, in which the normal samples were selected as the positive group and the parotid tumors as the negative group. As the result of the leave-one-sample-out method, 78 of 101 PA spectra, 78 of 105 WT spectra and 91 of 95 MEC spectra were classified correctly in the first step. According to the above results, the SVM could diagnoses the different spectra from the parotid tumors and normal samples successfully, and the parameters SP, SE, ACC, MCC and R of the SVM diagnostic results were shown in the Table 3. Then the diagnosis of different parotid tumor samples was also carried out by using SVM in the second step. The results showed that SVM achieved an acceptable performance on the classification of different parotid gland tumors. And the parameters SP, SE, ACC, MCC and R in the second step were shown in Table 4. And when the leave-one-patient-out method was applied, 58 of 101 PA spectra, 54 of 105 WT spectra and 61 of 95 MEC spectra were classified correctly in the first step. And the results of the classification by this method were shown in Tables 5 and 6.

**Table 3:** The parameters of the ‘Leave-one-sample’ classification results of the spectra from parotid tumor samples and normal samples using SVM

Parameter	Normal vs PA	Normal vs WT	Normal vs MEC
SP	77.2 %	74.3 %	73.7 %
SE	93.5 %	90.8 %	97.4 %
ACC	87.5 %	84.1 %	88.3 %
MCC	0.727	0.668	0.755
R	0.780	0.737	0.765

**Table 4:** The parameters of the ‘Leave-one-sample’ classification results of the spectra from the different parotid tumor samples using SVM

Parameter	PA vs WT	PA vs MEC	WT vs MEC
SP	86.7 %	82.1 %	86.3 %
SE	82.2 %	90.1 %	84.8 %
ACC	84.5 %	86.2 %	85.5 %
MCC	0.689	0.725	0.710
R	0.832	0.812	0.836

**Table 5:** The parameters of the ‘Leave-one-patient’ classification results of the spectra from parotid tumor samples and normal samples using SVM

Parameter	Normal vs PA	Normal vs WT	Normal vs MEC
SP	79.1 %	79.1 %	93.5 %
SE	57.4 %	51.4 %	64.2 %
ACC	70.5 %	67.8 %	82.3 %
MCC	0.371	0.328	0.621
R	0.663	0.639	0.862

**Table 6:** The parameters of the ‘Leave-one-patient’ classification results of the spectra from the different parotid tumor samples using SVM

Parameter	PA vs WT	PA vs MEC	WT vs MEC
SP	57.4 %	69.5 %	78.9 %
SE	57.1 %	68.3 %	67.6 %
ACC	57.3 %	68.9 %	73.0 %
MCC	0.159	0.389	0.473
R	0.256	0.555	0.684

## Discussion

In clinical examination, the blood samples are easily collected and mostly reflect some vital subtle change caused by tumors in the metabolism environment, such as amino acid metabolism, miRNA expression and biomarkers generation [32–34]. The concentration of nucleic acids and the composition of proteins from the serum and plasma samples of tumor patients are different from the normal samples, which are believed to originated from apoptosis, tumor necrosis and associated metabolites [22]. So in our exploratory study, blood serum detection is appropriate for the preoperative diagnosis of parotid gland tumors. It would be a revolution of tumor screening by using SERS to detect periphery blood samples for the preoperative diagnosis of parotid gland tumors.

In our study, the specific molecular differences of parotid gland tumors and normal serums were significantly demonstrated through the comparison between the various SERS spectra. These differences or alterations would be related with the proliferation and metabolism of tumors. In the comparison between the tumors and normal serums, the SERS peaks at around 723–727 cm^−1^ and 744–747 cm^−1^ assigned to the hypoxanthine and thymine in nucleic acids manifested the higher intensity in the parotid gland tumor groups, which could be resulted from the active metabolism of nucleic acids in the patients with parotid gland tumors. This result is also in agreement with the studies on the Raman spectra of parotid gland tumor tissues [2, 20]. The SERS peak at around 1326–1329 cm^−1^ was assigned to the CH vibration structure in nucleic acids, and the increased intensity of this peak in parotid gland tumor groups also suggested that there was an increased amount of nucleic acids in the serums from the patients with parotid gland tumors. This difference can be explained by the increased cell-free nucleic acids which originated from the apoptosis, necrosis and release of intact cells in the bloodstream and their subsequent lysis [16]. In the previous studies, the alteration of the nucleic acids in the tumorous blood samples could be detectable by SERS,so the SERS signal assigned to the nucleic acids in the serums can be employed as spectroscopic diagnostic biomarker to screen and monitor the occurrence of parotid gland tumors [15, 16, 18]. The SERS peaks at around 933 cm^−1^, 1084 cm^−1^, 1094 cm^−1^, 1368–1373 cm^−1^ and 1441–1445 cm^−1^ are attributed to the relative molecular structures in proteins. The higher intensities of these SERS peaks assigned to proteins in the parotid gland tumor serums demonstrated that there was an increase in the amount of relative proteins in the parotid gland tumor serums. Redistribution or translocation of plasma free amino acids in cancer patients was reported in the previous literature and the level of plasma free amino acids was related with the cancer type [32]. And the amount of these amino acids in plasma would increase in some cancers such as the breast cancer because these cancers does not grow as fast as the metabolically active cancers [32]. So these increased SERS peaks attributed to proteins in the serums can be employed as a diagnostic indicator to discriminate the parotid gland tumor serums from the normal ones. Meanwhile, there are some other differences existing in the SERS peaks of different parotid gland tumors, which may be due to the tumors’ various metabolisms and also can be considered as the diagnostic references. But compared with the serum SERS spectra reported in the other studies, some differences exist between the spectra in this study and the ones reported in the lietratures [29, 30]. These differences may result from the various nano-particles, the different preparation of serum and equipment parameters, and we could need a further research to find an exact explanation.

Based on the SERS spectra of the serum samples, an advanced algorithm is required to develop and establish an powerful diagnostic system. Numerous algorithms have been employed to analyze the Raman data such as principle component analysis (PCA), discrimination function analysis (DFA), partial least squares (PLS) and support vector machine (SVM). Compared with the other algorithms, SVM have been applied more extensively in many studies such as drug design, prediction of protein structure and diseases diagnosis due to its remarkable generalization performance [20, 35]. It is reported that SVM could classify and diagnose the oral squamous cell carcinoma based on the Raman spectra with the accuracy of approximate 98 % [25]. However, SVM is more powerful for the problem with small sampling, nonlinear and high dimension, and the increase of samples will waste more time and decrease the classification performance [35, 36, 37]. In this study, in order to reduce analytical errors, the process of classification by using SVM consisted of two step as reported in the previous study [20]. When the leave-one-sample-out method was applied for cross validation, the SERS spectra of tumorous serums and normal serums were classified and diagnosed successfully with an average accuracy of 86 %. But the accuracy of the classification carried out by the leave-one-patient-out cross validation decreased, the reason could be that only 4–6 independent spectra could not totally represent the differences in the serum of one patient. So in order to increase the diagnostic accuracy, the internal relation between the patient and spectrum should be explored and established in the further study. However, the classification results carried out by the two cross validation methods all manifested that WT was easier to be misdiagnosed as normal than MEC and PA probably due to the differences between benign and malignant tumors. Among the types of parotid gland tumors researched in this study, WT is benign tumor and MEC is malignant tumor. Although PA is benign tumor, it is considered as the critical tumor with the malignant degeneration potential [38]. So in the second step, MEC could be easier to be discriminated from PA and WT due to the diverse biological characters of the parotid gland tumors, which was also in agreement with the result in the study of Raman spectra of parotid gland tumor tissues [20]. But Beleites et al. demonstrate that the test sample sizes are necessary to achieve reasonable precision in the validation, too small sample size situation will completely mask the learning curve [39]. So this result of our preliminary study carried out by SVM could only demonstrate the potential to diagnoses and classify the different parotid gland tumors by SERS and SVM, and a further study involving a great number of samples should be needed. And the epidemiological characteristics of various tumors resulted in the differences and unbalances of the age and gender in the groups, which could interfere the diagnostic results of this study, it is also needed a large sample size experiment to give an explicit explanation.

## Conclusion

According to our knowledge, this is the first time to report that the SERS combined with SVM could be employed successfully to discriminate the serum samples of the patients with parotid gland tumors from the ones of the normal subjects and carry out a preoperative diagnosis of the parotid gland tumors. The differences existing in the SERS spectra of different samples manifested that the alterations of the biochemical metabolites in the serums from the patients with parotid gland tumors and normal control subjects. The serum SERS combined with SVM algorithm may have a giant potential to apply in the preoperative diagnosis and screening of parotid gland tumors if the further study could establish the relation between the spectra and patients.

## Abbreviations

SERS: Surface-enhanced Raman spectroscopy
NPs: Nanoparticles
SVM: Support vector machine
PL: Pleomorphic adenoma
WT: Warthin’s tumor
MEC: Mucoepidermoid carcinoma
SP: Specificity
SE: Sensitivity, ACC, accuracy
MCC: Matthew correlation coefficient
R: Rigidity
